# Long‐term trends and drought: Spatiotemporal variation in juvenile sex ratios of North American ducks

**DOI:** 10.1002/ece3.9099

**Published:** 2022-07-14

**Authors:** Sage L. Ellis, Madeleine G. Lohman, James S. Sedinger, Perry J. Williams, Thomas V. Riecke

**Affiliations:** ^1^ Department of Natural Resources and Environmental Science University of Nevada Reno Nevada USA; ^2^ Program in Ecology, Evolution, and Conservation Biology University of Nevada Reno Nevada USA; ^3^ Swiss Ornithological Institute Sempach Switzerland

**Keywords:** Bayesian, climate change, drought, hierarchical model, population sex ratio, sex‐specific survival, waterfowl

## Abstract

Sex ratios affect population dynamics and individual fitness, and changing sex ratios can be indicative of shifts in sex‐specific survival at different life stages. While climate and landscape changes alter sex ratios of wild bird populations, long‐term, landscape scale assessments of sex ratios are rare. Further, little work has been done to understand changes in sex ratios in avian communities. In this manuscript, we analyze long‐term (1961–2015) data on five species of ducks across five broad climatic regions of the United States to estimate the effects of drought and long‐term trends on the proportion of juvenile females captured at banding. As waterfowl have a 1:1 sex ratio at hatch, we interpret changes in sex ratios of captured juveniles as changes in sex‐specific survival rates during early life. Seven of 12 species‐region pairs exhibited evidence for long‐term trends in the proportion of juvenile females at banding. The proportion of juvenile females at banding increased for duck populations in the western United States and typically declined for duck populations in the eastern United States. We only observed evidence for an effect of drought in two of the 12 species‐region pairs, where the proportion of females declined during drought. As changes to North American landscapes and climate continue and intensify, we expect continued changes in sex‐specific juvenile survival rates. More broadly, we encourage further research examining the mechanisms underlying long‐term trends in juvenile sex ratios in avian communities.

## Graphical abstract

Assessments of changes in the drivers of population sex ratios are rare at landscape scales. We use a dataset of over 1.5m marked juvenile ducks to examine long‐term changes in sex ratio at banding over 55 years in the United States. Our results indicate an east‐west gradient in changes of juvenile sex ratios over time.

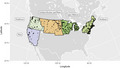

## 1 INTRODUCTION

Sex ratios are a key component of population structure, but are often assumed to be constant due to the difficulty of estimating sex ratios in wild populations (Lee et al., 2011). However, sex ratios often vary temporally and spatially in populations of wild organisms (Alisauskas et al., 2014; Fox & Cristensen, 2018; Frew et al., 2018; Lemons et al., 2012). This variation can influence population dynamics and may indicate changes in ecological processes that influence sex ratios either pre‐ or post‐nascence. Further, population sex ratios and mating systems are important drivers of effective population size, demographic stochasticity, and extinction risk (Bessa‐Gomes et al., 2004; Lee et al., 2011; Nunney, 1993). Thus, understanding population sex ratios is critical for the development of ecological theory and successfully applied conservation efforts (Donald, 2007; Mayr, 1939).

Reviews of sex ratios in birds (Donald, 2007; Mayr, 1939) have repeatedly demonstrated that for the majority of bird populations, sex ratios differ from equilibrium and are male biased. Researchers have identified three primary drivers behind skewed sex ratios. First, skewed sex ratios can arise due to unequal sex ratios at fertilization or conception. For instance, climatic variation has been linked to a skewed sex ratio in juvenile red‐winged blackbirds (*Agelaius phoeniceus*), where longer nesting seasons lead to maternal adjustment of offspring sex ratio favoring juvenile females (Weatherhead, 2005). Similarly, Seychelles warblers (*Acrocephalus sechellensis*) facultatively adjust the sex ratio of eggs before laying based on the quality of habitat, with higher quality habitat favoring female‐biased sex ratios (Komdeur, 1996; Komdeur et al., 1997). Second, differing sex ratios at birth might cause skewed sex ratios in a population, potentially due to sex‐specific hatching probability in birds (Donald, 2007). Third, variation in sex‐specific mortality rates could lead to skewed sex ratios in juveniles and adults (Eberhart‐Phillips et al., 2018; Gownaris & Boersma, 2019; Veran & Beissinger, 2009). For instance, a number of studies have shown that variation in resource requirements of different sexes can lead to variation in juvenile mortality rates (Cooch et al., 1997; Lemons et al., 2012; Weatherhead & Montgomerie, 1995). However, despite this body of research, few studies have examined variation in juvenile sex ratios of avian communities at continental or even regional scales (but see Fox & Cristensen, 2018).

North American waterfowl banding data provide a rich opportunity to examine ecological questions at broad spatiotemporal scales (e.g., Ross et al., 2015; Specht & Arnold, 2018; Zhao et al., 2019). As early as 1933, Aldo Leopold commented that the sex ratio of ducks was “seriously deranged” (Leopold, 1933) while reviewing data generated by Lincoln (1932) that indicated duck populations have exceedingly male‐biased adult sex ratios (Bellrose et al., 1961; Mayr, 1939). Puzzlingly, there is substantial evidence that waterfowl have an approximately even sex ratio at hatch (Bellrose et al., 1961; Blums & Mednis, 1996; Clutton‐Brock, 1986; Cooch et al., 1997; Hepp et al., 1989; Lehikoinen et al., 2008; Lemons et al., 2012; Swennen et al., 1979). In ducks, the skewed adult sex ratio is driven by increased mortality risk for adult females during the breeding season. While males invest energy in plumage (Promislow et al., 1994) and mating attempts, females must produce, lay, and incubate eggs and then brood and defend ducklings for several weeks. This energetic expenditure toward reproduction and increased predation risk (Sargeant et al., 1984) leads to reduced survival of breeding females relative to males (Arnold et al., 2016) and skews adult sex ratios. However, the potential for sex‐biased survival during early life remains underexamined for ducks and other wildlife species, particularly at broad spatiotemporal scales. Sex‐biased survival during early life can have important implications. For example, biased offspring sex ratios due to sex‐specific juvenile survival rates in common eiders (*Somateria mollissima*), snowy plovers (*Charadrius nivosus*), and Magellanic penguins (*Spheniscus magellanicus*) lead to biased adult sex ratios in these species (Eberhart‐Phillips et al., 2018; Gownaris & Boersma, 2019; Lehikoinen et al., 2008). Given a dramatic increase in adult male to female ratios in North American duck populations (Alisauskas et al., 2014; Arnold et al., 2017) and the potential for variation in sex‐specific juvenile mortality to affect adult sex ratios, we sought to examine long‐term trends in sex ratios of juvenile ducks in North America.

**FIGURE 1:**
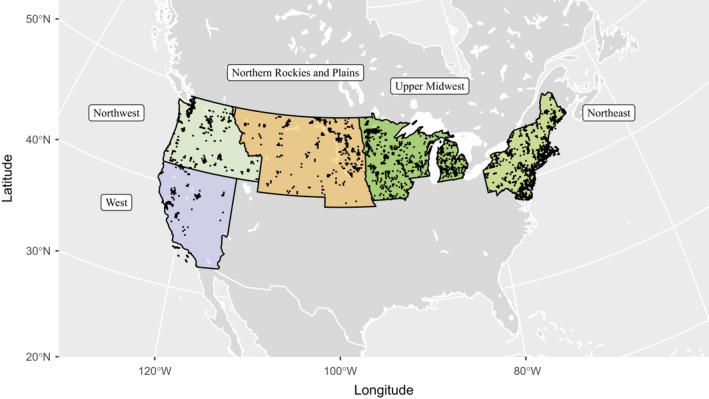
Release locations (*n* = 2291) of 1,578,169 hatch‐year ducks of five species marked in the West, Northwest, Northern Rockies and Plains, Upper Midwest, and Northeast U.S. Climate Regions (Karl & Koss, 1984) from 1961 to 2015

We formulated two research questions regarding the sex ratio of juvenile ducks in North America: (1) Is the decline in the proportion of juvenile female ducks at banding in North America similar to declines observed in Europe (Fox & Cristensen, 2018), and do these declines vary spatially or among species? (2) Given projected changes in precipitation and the strong linkage between hydrologic conditions and waterfowl populations (Sorenson et al., 1998), does drought play a role in affecting the sex ratio of juvenile ducks? We addressed these questions by modeling the effects of the regional Palmer Hydrological Drought Index (PHDI) and long‐term trends on the proportion of juveniles captured at banding that were female for five duck species in five climatic regions defined by the National Center for Environmental Information (Figure 1; Karl & Koss, 1984) from 1961 to 2015.

## 2 METHODS

We downloaded capture‐release data for five different species of ducks that occur in the contiguous United States: mallards (*Anas platyrhynchos*), northern pintails (*Anas acuta*), blue‐winged teal (*Anas discors*), American black ducks (*Anas rubripes*), and wood ducks (*Aix sponsa*) from the U.S. Geological Survey GameBirds CD (Patuxent, MD, USA; USGS Bird Banding Laboratory, 2017) for the years 1961–2015. We restricted release data to birds that were marked as hatch‐year (i.e., flighted juvenile) from July to September. We obtained Palmer Hydrological Drought Index values from the National Oceanic and Atmospheric Administration's Climate at a Glance: Regional Time Series from January 1961 to December 2015 (NOAA, 2020) and used the mean PHDI value from May to June as a measure of drought during the breeding season. We chose the Upper Midwest, Northeast, Northwest, West, and Northern Rockies and Plains climatic regions as study areas as these are the primary breeding areas for ducks in the contiguous United States (Figure 1; Karl & Koss, 1984).

We partitioned the release data into these five climate regions. Total releases for each species in each region are provided in Table 1, and sample sizes through time per region, species, and sex can be found in the Supplemental material (S1). We only included species and regions in analyses when greater than 27,500 individuals (500 individuals/year, on average) had been released over the course of the study in a region. Since not all species were well represented in all regions across time, bold values in Table 1 represent the species‐region pairs that we analyzed in this manuscript.

**TABLE 1:** Total hatch‐year (i.e., flighted juvenile) captures of American black duck (ABDU), blue‐winged teal (BWTE), mallard (MALL), northern pintail (NOPI), and wood duck (WODU) in each NOAA U.S. Climate Region (Karl & Koss, 1984) from July–September 1961–2015

Region	Species codes				
	ABDU	BWTE	MALL	NOPI	WODU
Northeast	**70,962**	**28,529**	**201,496**	1833	**122,183**
Upper Midwest	8498	**59,545**	**338,093**	5126	**182,944**
Northern Rockies and Plains	23	**173,925**	**145,877**	**45,814**	2408
Northwest	0	748	**116,309**	10,219	8978
West	0	13	**91,289**	15,002	1964

*Note:* Capture and release data are from the U.S. Geological Service Bird Banding Laboratory GameBirds CD (Patuxent, MD, USA). Bold values indicate the specific species and regions used in the analysis.

We developed a Bayesian hierarchical model that we describe using the hierarchical convention of Berliner (1996). We first created a data model which links our data, total captures of juvenile females and males per year in each region, to our proposed ecological process model for the probability of each juvenile being a female. Probability density or mass functions of our variables are noted using square brackets, so that [*a*|*b*] represents the probability distribution of random variable *a* conditional on *b*. We used the same model for each species‐region pair.

For each species‐region pair, we estimated the probability of a juvenile being female during each year (*π*_t_) as a function of the number of captured juvenile females (*y*_t_) and the total number of captured juveniles (*η*_t_) using a binomial distribution,

(1) $$y_{t}\sim \text{Binomial}\left(\eta_{t},\pi_{t}\right)$$

We modeled the log odds of the probability of a juvenile being female using a normal distribution with a time‐varying mean (*μ*_t_) with uncertainty (*σ* ^2^). We modeled the time‐varying mean as a function of an intercept (*α*), the Palmer Hydrological Drought Index (PHDI) specific to each climate region during each year, and a long‐term trend.

(2) $$\begin{array}{l} \text{logit}\left(\pi_{t}\right)\sim \text{Normal}\left(\mu_{t},\sigma^{2}\right),\\ \mu_{t}=\alpha +\beta_{\text{PHDI}}\times {\text{PHDI}}_{t}+\beta_{\text{time}}\times t. \end{array}$$

We assumed PHDI was measured without error. Negative PHDI values indicate drought, while positive values indicate wet years. Thus, when interpreting *β*_PHDI_ values, a positive value indicates that the proportion of juvenile females at banding decreases during drought and a negative value indicates that the proportion of juvenile females at banding increases during drought. Each parameter included in the data and process model above required a prior distribution. We chose vague priors for each parameter,

(3) $$\begin{array}{l} \sigma \sim \text{gamma}\left(1,1\right),\\ \alpha \sim \text{normal}\left(0,2.25\right),\\ \beta \sim \text{normal}\left(0,10\right), \end{array}$$

where ***β*** = (*β*_PHDI_, *β*_time_). The joint posterior distribution of the parameters, given the data, is,

(4) $$\left[\alpha ,\beta ,\sigma ,\pi \left|y\right.,\eta \right]\propto \prod_{t=1}^{T}\left[y_{t}|\;\pi_{t},\eta_{t}\right]\left[\pi_{t}|\alpha ,\beta ,\sigma \right]\times \left[\alpha \right]\left[\beta \right]\left[\sigma \right].$$

We used the package rjags (Plummer, 2019) in R version 3.6.2 (R Core Team, 2019) for our analysis. We sampled three chains for 1,000,000 iterations, with a burn‐in of 500,000 iterations. We retained every 50th iteration to avoid memory and storage limitations. We visually assessed the convergence of parameters using trace plots, and all parameters had $\hat{R}<1.01$ for all models (Gelman & Rubin, 1992). We used posterior predictive checks to calculate Bayesian *p*‐values for model checking, using the deviance discrepancy function as described in Conn et al. (2018). Bayesian *p*‐values were between .1 and .9 for all models. Thus, our model checking shows no evidence for lack of fit (Table 2).

**TABLE 2:** Bayesian *p*‐values of models estimating the proportion of hatch‐year (i.e., flighted juvenile) ducks that are female for American black duck (ABDU), blue‐winged teal (BWTE), mallard (MALL), northern pintail (NOPI), and wood duck (WODU) in five NOAA U.S. Climate Regions (Karl & Koss, 1984) from 1961 to 2015

Region	Species codes				
	ABDU	BWTE	MALL	NOPI	WODU
Northeast	0.72	0.42	0.53	–	0.63
Upper Midwest	–	0.88	0.52	–	0.49
Northern Rockies and Plains	–	0.80	0.58	0.72	–
Northwest	–	–	0.54	–	–
West	–	–	0.59	–	–

## 3 RESULTS

**FIGURE 2:**
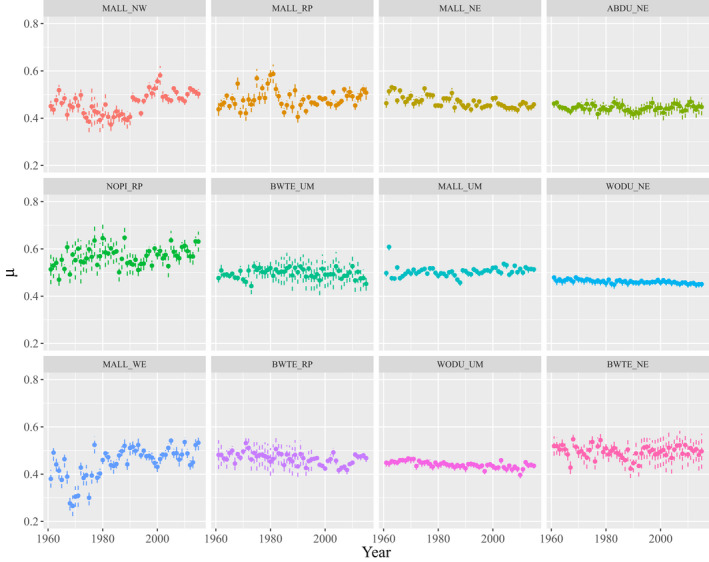
Means (points) and 95% credible intervals (dashed lines) of posterior distributions for estimates of the proportion of females for American black duck (ABDU), blue‐winged teal (BWTE), mallard (MALL), northern pintail (NOPI), and wood duck (WODU) populations in the Northeast (NE), Northern Rockies and Plains (RP), Upper Midwest (UM), Northwest (NW), and West (WE) U.S. Climate Regions (Karl & Koss, 1984)

**FIGURE 3:**
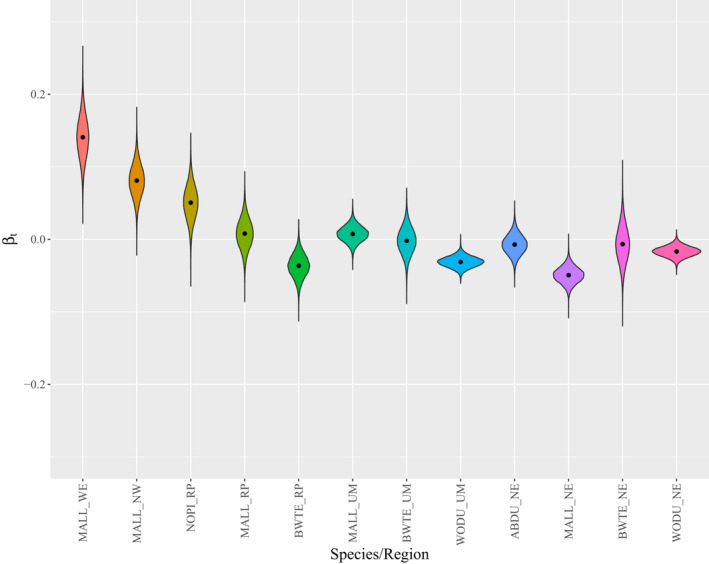
Violin plots of marginal posterior distributions and means (points) of the effect of long‐term trends on the proportion of juvenile females for American black ducks (ABDU), blue‐winged teal (BWTE), mallards (MALL), northern pintails (NOPI), and wood ducks (WODU) in the Northeast (NE), Northern Rockies and Plains (RP), Upper Midwest (UM), Northwest (NW), and West (WE) U.S. Climate Regions (Karl & Koss, 1984). Effects greater than 0 indicate long‐term increases in the proportion of juveniles that are female, and effects less than 0 indicate long‐term declines in the proportion of juveniles that are female

**FIGURE 4:**
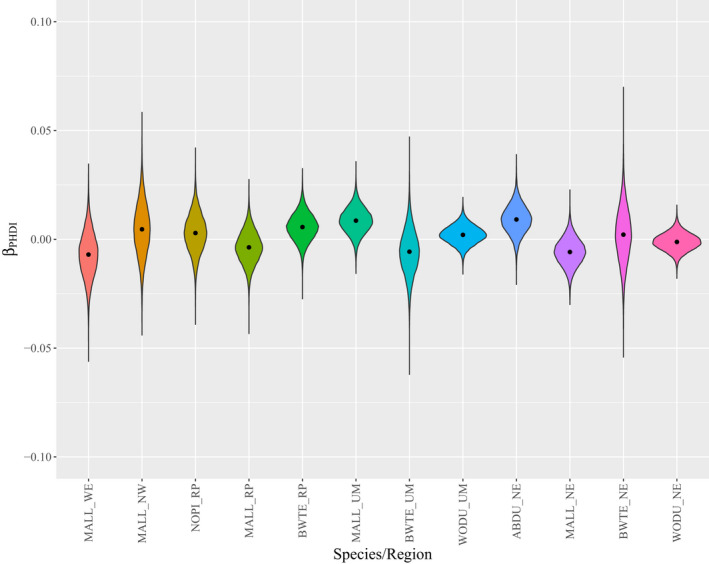
Violin plots of marginal posterior distributions and means (points) of the effect of the Palmer Hydrological Drought Index (PHDI) on the proportion of juvenile female American black ducks (ABDU), blue‐winged teal (BWTE), mallards (MALL), northern pintails (NOPI), and wood ducks (WODU) in the Northeast (NE), Northern Rockies and Plains (RP), Upper Midwest (UM), Northwest (NW), and West (WE) U.S. Climate Regions (Karl & Koss, 1984). Effects greater than 0 indicate that drought decreases the proportion of juveniles that are female, and effects less than 0 indicate drought increases the proportion of juveniles that are female

Greater than 1.5 million hatch‐year ducks were captured at 2291 unique sites (Figure 1) across five different U.S. climate regions from 1961 to 2015 (Table 1). We found support for long‐term changes in juvenile sex ratios for 7 of 12 species‐region pairs (Figure 2), but only observed drought effects in two species‐region pairs. The direction of temporal trends varied among species and regions, but seemed to generally vary along a longitudinal gradient (Figure 3), where sex ratios became more female biased in western regions and more male biased in eastern regions. Mallard populations experienced long‐term increases in the proportion of juvenile females at banding from 0.42 in 1961 to 0.50 in 2015 in the Northwest (*β*_time_ = 0.081, *f* = 1), and from 0.38 to 0.52 over the same time period in the West (*β*_time_ = 0.141, *f* = 1) climate regions. In the Northern Rockies and Plains climate region, the proportion of juvenile females at banding increased from 0.54 to 0.59 for northern pintail (*β*_time_ = 0.051, *f* = 0.98), was stable for mallard, and declined from 0.48 to 0.45 for blue‐winged teal (*β*_time_ = −0.037, *f* = 0.99) over the course of the study. In the Upper Midwest climate region, the proportion of juvenile females at banding declined from 0.46 to 0.42 for wood duck (*β*_time_ = −0.031, *f* = 1), but was stable for mallard and blue‐winged teal (Table 3). Finally, the proportion of juvenile females at banding declined from 0.49 to 0.45 for mallard (*β*_time_ = −0.049, *f* = 1), and from 0.47 to 0.45 for wood duck (*β*_time_ = −0.017, *f* = 0.99) in the Northeast climate region, but was stable for blue‐winged teal and American black duck. Drought generally did not impact sex ratios of juvenile waterfowl (Table 3), where there was little support for variation in the proportion of females at banding as a function of the Palmer Hydrological Drought Index (Figure 4) for most species‐region pairs. However, there was evidence that drought led to lower proportions of juvenile females at banding for American black duck in the Northeast climate region (*β*_time_ = 0.009, *f* = 0.92), and mallards in the Upper Midwest climate region (*β*_time_ = 0.009, *f* = 0.93).

**TABLE 3:** Means (*μ*), standard deviations (*σ*), and *f*‐values (*f*; the proportion of the marginal posterior distribution on the same side of zero as the mean) of regression parameter posterior distributions for the effects of a long‐term trend (Time) and Palmer Hydrological Drought Index (PHDI) from models of juvenile sex ratio of American black duck (ABDU), blue‐winged teal (BWTE), mallard (MALL), northern pintail (NOPI), and wood duck (WODU) marked in five NOAA U.S. Climate Regions (NE = Northeast, UM = Upper Midwest, RP = Northern Rockies and Plains, NW = Northwest, and WE = West; Karl & Koss, 1984) from 1961–2015

Species_Region	Time			PHDI		
	*μ*	*σ*	*f*	*μ*	*σ*	*f*
ABDU_NE	−0.007	0.014	0.71	0.009	0.006	0.92
BWTE_UM	−0.002	0.018	0.54	−0.006	0.010	0.73
BWTE_RP	−0.037	0.016	0.99	0.006	0.006	0.82
BWTE_NE	−0.007	0.025	0.61	0.002	0.012	0.58
MALL_NE	−0.049	0.011	1	−0.006	0.006	0.83
MALL_UM	0.007	0.011	0.76	0.009	0.006	0.93
MALL_RP	0.008	0.020	0.65	−0.004	0.007	0.70
MALL_NW	0.081	0.023	1	0.005	0.012	0.65
MALL_WE	0.141	0.029	1	−0.007	0.010	0.76
NOPI_RP	0.051	0.024	0.98	0.003	0.009	0.63
WODU_NE	−0.017	0.007	0.99	−0.001	0.004	0.62
WODU_UM	−0.031	0.007	1	0.002	0.004	0.69

## 4 DISCUSSION

We observed substantial evidence for long‐term changes in the proportion of juvenile females at banding relative to juvenile males in seven of the 12 study populations. Strikingly, this effect appears to change across longitudinal gradients, where the proportion of juvenile females at banding increased in western populations and declined in eastern populations, similar to declines observed in Europe (Fox & Cristensen, 2018). Drought effects on sex ratios were rarely observed (two of 12 study populations), but consistently led to a lower proportion of juvenile females at banding during drought years when effects were significant. Numerous studies have documented 50:50 waterfowl sex ratios at hatch (e.g., Blums & Mednis, 1996; Lemons et al., 2012). Thus, we interpret shifts in the proportion of juvenile females at banding as evidence for sex‐specific variation in survival during early life, but cannot confirm any driving force.

We observed inter‐regional variation in long‐term changes in the proportion of juvenile females at banding. There are numerous hypotheses that might explain species‐ and region‐specific variation in long‐term trends in the proportion of juvenile females at banding. Lesser snow goose (*Anser caerulescens caerulescens*) juvenile sex ratios at banding became more female biased over time as habitat degraded because of an overabundance of geese in breeding areas (Cooch et al., 1997). Cooch et al. (1997) attributed this shift to higher juvenile male mortality due to resource reduction, and the increased energetic requirements of structurally larger male goslings. Lemons et al. (2012) drew similar conclusions for a female‐biased juvenile sex ratio due to differential early‐life mortality in black brant (*Branta bernicla nigricans*). Thus, one hypothesis is that perhaps these long‐term changes in the proportion of females at banding may be due to long‐term shifts in habitat quality that vary longitudinally.

The species‐region pairs with evidence for drought effects had lower proportions of juvenile females at banding during drier years and lower proportions of juvenile males at banding during wetter years. This is in direct contrast to the resource limitation hypothesis; drought conditions reduce available food resources, leading to decreased early‐life survival rates for larger‐bodied individuals (i.e., males) relative to females. Of note, none of the species modeled in the Northern Rockies and Plains (i.e., Prairie Pothole Region) had support for drought affecting the proportion of juvenile females at banding, despite large sample sizes and clear evidence of drought affecting other duck demographic rates (Dufour & Clark, 2002; Specht & Arnold, 2018; Walker et al., 2013). Future research should examine differential relationships between environmental conditions and sex‐specific duckling survival at finer scales.

While our results provide insights into long‐term changes at landscape scales, unexplained heterogeneity undoubtedly exists within the climatic regions examined in this study. For example, within the Prairie Pothole Region, located in the Northern Rockies and Plains, western portions are becoming drier with less wetland availability, while eastern portions become wetter with more wetland availability (Millett et al., 2009; Niemuth et al., 2014). Substantial heterogeneity in fecundity also occurs at finer scales within the broad climatic regions we used as study areas (Specht & Arnold, 2018). Thus, perhaps our analyses did not adequately capture the effects of climate by using PHDI at large regional scales. We note that sampling effort, intensity, and location might also affect our results. For instance, during drought years, field biologists trap ducks in extant wetlands that may have different habitat quality and conditions than nearby dry ephemeral or semi‐permanent wetlands. Further, field biologists sometimes band to meet local age‐ and sex‐class‐specific quotas. This might induce additional heterogeneity into the mark‐release data. Density‐dependent mechanisms may also impact sex‐specific juvenile duckling survival, and the duration and extent of existing abundance surveys did not allow us to incorporate the effects of density‐dependence across all regions. These density‐dependent mechanisms may vary spatially (Specht & Arnold, 2018; Zhao et al., 2016) and may act interactively with climate change to affect future waterfowl demographic rates (Zhao et al., 2018).

We might expect continued long‐term changes in juvenile duck sex ratios as climate and anthropogenic actions continue to impact these broad regions. As population demographers move away from including sex ratio as a constant in population estimates, small changes in sex ratio may influence projections from population models and associated management actions. It will be of paramount importance to continue banding efforts to monitor long‐term changes in sex ratio and other demographic rates. While we have briefly discussed potential explanations for long‐term trends, the underlying mechanistic reasons for the observed patterns in the data were not tested in our analyses and should be examined further. Given the rarity at which researchers have examined changes in juvenile sex ratios at broad scales (Fox & Cristensen, 2018) in avian communities, we encourage continued research to estimate baseline juvenile sex ratios in avian communities and examine the mechanisms underlying long‐term trends and short‐term variation in response to climatic anomalies and other perturbations.

## AUTHOR CONTRIBUTIONS

**Sage L. Ellis:** Conceptualization (equal); data curation (lead); formal analysis (lead); writing – original draft (lead); writing – review and editing (equal). **Madeleine G. Lohman:** Conceptualization (equal); formal analysis (supporting); writing – original draft (supporting); writing – review and editing (supporting). **James S. Sedinger:** Conceptualization (equal); writing – original draft (supporting); writing – review and editing (supporting). **Perry J. Williams:** Conceptualization (supporting); formal analysis (equal); writing – original draft (supporting); writing – review and editing (supporting). **Thomas V. Riecke:** Conceptualization (equal); formal analysis (equal); writing – original draft (supporting); writing – review and editing (equal).

## Supporting information


Supplementary material

## Data Availability

The data used in this study are compiled and publicly available through the USGS Bird Banding Laboratory (duck banding data; Celis‐Murillo et al., 2020) and NOAA (PHDI data). Code used to analyze the data is provided as Supplementary material.
