# Supplementary material for: Long‐term trends and drought: Spatiotemporal variation in juvenile sex ratios of North American ducks
Source: Ecol Evol. 2022 Jul 14;12(7):e9099. doi: 10.1002/ece3.9099 (PMC9280441; doi:10.1002/ece3.9099)
Supplement: Supplementary file 1 — Supplementary material [file ECE3-12-e9099-s001.pdf]

Sex ratios of juvenile North American ducks: long-term trends and drought

Sage L. Ellis[1], Madeleine G. Lohman[1,2], James S. Sedinger[1], Perry J. Williams[1], and Thomas V. Riecke[1,2,3]

[1]Department of Natural Resources and Environmental Science, University of Nevada, Reno, NV 89557, USA
[2]Program in Ecology, Evolution, and Conservation Biology, University of Nevada, Reno, NV 89557, USA
[3]Swiss Ornithological Institute, 6204 Sempach, Switzerland

Contents

1 Data **1**
1.1 PHDI 1
1.2 Release Data 3
1.3 Total Releases 5

2 Model Statement **6**
2.1 Data Model 6
2.2 Process Model 6
2.3 Parameter Model 7

3 Joint Posterior Distribution **7**

4 Full Conditional Distribution **7**

5 Model Code **7**

6 Figures **11**
6.1 Release Plots 11
6.2 Sex Ratio Plots 22

1 Data

1.1 PHDI

We downloaded PHDI data from `https://www.ncdc.noaa.gov/cag/regional/time-series` at 1-month intervals from January 1961 - January 2016 for the

Upper Midwest, Northeast, Northwest, West, and Northern Rockies and Plains Climate Regions.

To shape PHDI data:

```
##################
#Group PHDI Values
##################

###
#Data aquired from:
#https://www.ncdc.noaa.gov/cag/regional/time-series
###

#Required package
library(tidyr)

#Read in your raw PHDI data
NE_PHDI <- read.csv(paste0(dir, files[1]))
WE_PHDI <- read.csv(paste0(dir, files[2]))
UM_PHDI <- read.csv(paste0(dir, files[3]))
RP_PHDI <- read.csv(paste0(dir, files[4]))
NW_PHDI <- read.csv(paste0(dir, files[5]))

seperate <- function(df){
  df[["Month"]] <- substr(df[["Date"]], 5,6)
  df[["Year"]] <- substr(df[["Date"]], 1, 4)
  df <- df[,c("Year", "Month", "Value")]
  df <- spread(df, Month, Value)
  colnames(df)[2:13] <- month.name
  return(df[-56,])
}

#Reshape data
NE_PHDI <- seperate(df = NE_PHDI)
WE_PHDI <- seperate(df = WE_PHDI)
UM_PHDI <- seperate(df = UM_PHDI)
RP_PHDI <- seperate(df = RP_PHDI)
NW_PHDI <- seperate(df = NW_PHDI)

summs <- function(df){
  brood <- month.name[4:8]  #Brood Rearing Months: April-Aug
  df$Brood <- rowMeans(df[,which(colnames(df) %in% brood)],
                       na.rm = T)
  return(df)
}
```

```r
#Take average PHDI value from April-August
NE_PHDI <- summs(df = NE_PHDI)
WE_PHDI <- summs(df = WE_PHDI)
UM_PHDI <- summs(df = UM_PHDI)
RP_PHDI <- summs(df = RP_PHDI)
NW_PHDI <- summs(df = NW_PHDI)
```

1.2 Release Data

We requested release data from the U.S. Geological Service Bird Banding Laboratory GameBirds CD (Patuxent, MD, USA) at https://www.usgs.gov/centers/pwrc/science/banding-and-encounter-data-requests?qt-science_center_objects=0#qt-science_center_objects for American black ducks (abdu), blue-winged teal (bwte), mallards (mall), northern pintail (nopi), and wood ducks (wodu) from 1961-2016.

To shape release data:

```r
###
#Read in your release data as: abdu_rel; bwte_rel; mall_rel;
#                              nopi_rel; and wodu_rel
###

#Delineate climate regions into their respective states

#Upper Midwest
UM <- c("Iowa","Michigan","Minnesota", "Wisconsin")

#Northeast
NE <- c("Connecticut","Delaware","Maine","Maryland",
        "Massachusetts","New Hampshire", "New Jersey","New York",
        "Pennsylvania","Rhode Island","Vermont")

#Northwest
NW <- c("Idaho", "Oregon", "Washington")

#West
WE <- c("California", "Nevada")

#Northern Rockies and Plains
RP <- c("Montana", "Nebraska", "North Dakota", "South Dakota",
        "Wyoming")

speciesdf <- list(abdu_rel, bwte_rel, mall_rel,
```

```
                  nopi_rel, wodu_rel)

reg <- list(UM, NE, NW, WE, RP)

start <- 1961
end <- 2016

#Pull juveniles from release data based on the species and region
for(i in 1:length(speciesdf)){
  for(j in 1:length(regions)){
    #Subset only states in our climate regions
    x <- subset(speciesdf[[i]], STATE_NAME %in% reg[[j]])
    #Subset data to only include 1961-2016
    releases <- subset(x, B.Year >= start & B.Year < end)
    #Subset to include only normal wild birds(3)
    releases <- subset(releases, Status == 3)
    #Subset to only include Hatch Year(2) and Local birds(4)
    releases <- subset(releases, Age == 2 | Age == 4)
    #Subset to only include data from our brood rearing months
    #   (April-August)
    releases <- subset(releases, B.Month >= 4 & B.Month <= 8)
    summary(releases$SPEC)
    releases <- subset(releases, SPEC == toupper(species[i]))
    releases <- subset(releases,
                       VAI == 'Captured by spotlighting.' |
                       VAI ==
                      'Control band (Reward band studies only).' |
                       VAI == 'Federal numbered metal band only.')
    summary(releases$VBtype)
    releases <- subset(releases, VBtype == 'Aluminum butt-end' |
                       VBtype == 'aluminum butt-end ' |
                       VBtype == 'Aluminum pre-open' |
                       VBtype == 'Incoloy/SS butt end' |
                       VBtype == 'Monel butt end')
    print(sum(releases$Count.of.Birds))
    assign(paste0(species[i], "_",regions[j]),releases)
  }
}

abdu_reg <- list(abdu_UM = abdu_UM, abdu_NE = abdu_NE,
                 abdu_NW = abdu_NW, abdu_WE = abdu_WE,
                 abdu_RP = abdu_RP)

bwte_reg <- list(bwte_UM = bwte_UM, bwte_NE = bwte_NE,
                 bwte_NW = bwte_NW, bwte_WE = bwte_WE,
```

```
                  bwte_RP = bwte_RP)

mall_reg <- list(mall_UM = mall_UM, mall_NE = mall_NE,
                 mall_NW = mall_NW, mall_WE = mall_WE,
                 mall_RP = mall_RP)

nopi_reg <- list(nopi_UM = nopi_UM, nopi_NE = nopi_NE,
                 nopi_NW = nopi_NW, nopi_WE = nopi_WE,
                 nopi_RP = nopi_RP)

wodu_reg <- list(wodu_UM = wodu_UM, wodu_NE = wodu_NE,
                 wodu_NW = wodu_NW, wodu_WE = wodu_WE,
                 wodu_RP = wodu_RP)
```

1.3 Total Releases

Calculate total releases and release plots over time for each species_region.

```
start <- 1961
end <- 2016

rgns <- c('UM','NE','NW','WE','RP')
regions <- c("Upper Midwest", "Northeast", "Northwest", "West",
             "Northern Rockies and Plains")
species <- c("abdu", "bwte", "mall", "nopi", "wodu")

total_rel <- data.frame(matrix(nrow = length(regions),
                         ncol = length(species)))
colnames(total_rel) <- species
rownames(total_rel) <- regions

for(s in 1:length(species)){
  #Format Data
  spec <- get(paste0(species[s], '_reg'))

  data <- array(NA, dim = c(55, 5, 5))
  data[,4,] <- seq(1,55)
  data[,5,] <- seq(start,(end-1))

  for (i in 1:55){
    for (j in 1:5){
      data[i,1,j] <- sum(spec[[j]]$Count.of.Birds[which(
                         spec[[j]]$B.Year == (i + 1960) &
                         spec[[j]]$Sex == 5)])
      data[i,2,j] <- sum(spec[[j]]$Count.of.Birds[which(
```

```
                         spec[[j]]$B.Year == (i + 1960) &
                         spec[[j]]$Sex == 4)])
      data[i,3,j] <- data[i,1,j] + data[i,2,j]
      assign(names(spec[j]), data[,,j])
    }
  }

  for(j in 1:length(rgns)){
    totals <- get(paste0(species[s], '_', rgns[j]))
    total_rel[j,s] <- sum(totals[,3])
  }

  #Release Plots
  par(mfrow=c(3,2),family='serif')
  for(i in 1:length(rgns)){
    dat <- get(paste0(species[s],'_',rgns[i]))

    barplot(dat[,c(1,2)]~ seq(1961,2015), ylab = 'Releases',
            xlab = 'Year', beside = T,
            col = c("#FF8C13", "#1386FF"),
            main = paste(toupper(species[s]), "-", regions[i]))
  }
}

total_rel
```

2 Model Statement

2.1 Data Model

$$y_t \sim \text{binomial}(\eta_t, \pi_t)$$

where y_t is the total number of females in a given year, t, in a given region, r. The total number of individuals in a given year and region is represented by η_t and π_t denotes the probability of being female for a given year and region.

2.2 Process Model

$$\begin{aligned}\text{logit}(\pi_t) &\sim \text{normal}(\mu_t, \sigma^2)\\ \mu_t &= \alpha + (\beta_{PHDI} * \text{PHDI}_t) + (\beta_t * t)\end{aligned}$$

where π_t denotes the probability of being female for a given year, t and region, r. This is derived from the mean probability of being female for each region and year, μ_t and some uncertainty, σ^2. Our model assumed that the probability of being female is influenced by some true value for the proportion of females in the population for each region, α, which is altered based on drought ($\beta_{PHDI} * \text{PHDI}_t$), time ($\beta_t * t$), and the interaction between the two ($\beta_{int} * \text{PHDI}_t * t$).

2.3 Parameter Model

$$\begin{aligned}
\sigma &\sim \text{uniform}(0, 3)\\
\alpha &\sim \text{normal}(0, 2.25)\\
\boldsymbol{\beta} &\sim \text{normal}(0, 10)
\end{aligned}$$

3 Joint Posterior Distribution

$$\begin{aligned}
[\alpha, \boldsymbol{\beta}, \sigma, \boldsymbol{\pi}|\boldsymbol{y}] \propto \prod_{t=1}^{T}&[y_t|\pi_t, N_t][\pi_t|\mu_t, \sigma]\\
&\times\ [\mu_t|\alpha, \boldsymbol{\beta}]\\
&\times\ [\alpha][\boldsymbol{\beta}][\sigma]
\end{aligned}$$

4 Full Conditional Distribution

$$\begin{aligned}
[\alpha|.] &\propto \prod_{t=1}^{T}[\mu_t|\alpha, \boldsymbol{\beta}][\alpha]\\
[\boldsymbol{\beta}|.] &\propto \prod_{t=1}^{T}[\mu_t|\alpha, \boldsymbol{\beta}][\boldsymbol{\beta}]\\
[\sigma|.] &\propto \prod_{t=1}^{T}[\pi_t|\mu_t, \sigma][\sigma]\\
[\pi|.] &\propto \prod_{t=1}^{T}[y_t|\pi_t, N_t][\phi_t|\mu_t, \sigma]
\end{aligned}$$

5 Model Code

```
require(jagsUI)
# Specify model in BUGS language
sink("age.jags")
cat("
    model {

    ######################
    # Priors
    ######################

    alpha ~ dnorm(0, 0.4)
    beta.PHDI ~ dnorm(0, 0.1)
    beta.time ~ dnorm(0, 0.1)
    beta.int ~ dnorm(0, 0.1)
    sig ~ dunif(0,3)
    tau <- pow(sig, -2)


    for(t in 1:n.years){

      ######################
      # Process
      ######################

      mu[t] = alpha + (beta.PHDI * PHDI[t]) + (beta.time * t)

      ######################
      # Sampling
      ######################

      eps[t] ~ dnorm(mu[t], tau)
      logit(rat[t]) <- eps[t]

      ######################
      # Observation
      ######################

      dat[t,1] ~ dbin(rat[t], dat[t,3]) #Observed data
      y.ppd[t] ~ dbin(rat[t], dat[t,3]) #Simulated data

    }

    dummy ~ dbeta(1,1)

    }
```

```
    ",fill = TRUE)
sink()

###
#Run model for each species in each region
###

for(s in 1:length(species)){
  for(r in 1:length(rgns)){
    rgn_PHDI <- get(paste0(rgns[r],"_PHDI"))
    species_rgn <- get(paste0(species[s], "_", rgns[r]))

    jags.data <- list(n.years = 55,
                      PHDI = as.numeric(rgn_PHDI[,15]),
                      dat = species_rgn
    )

    # Initial values
    inits <- function(){list(dummy = 0.5)}

    # Parameters monitored
    parameters <- c('rat','beta.PHDI','beta.time','beta.int',
                    'alpha', 'sig')

    # MCMC settings
    ni <- 30000
    nt <- 5
    nb <- 10000
    nc <- 3


    # Call JAGS

    m <- jags(jags.data, inits, parameters, "age.jags",
              n.chains = nc, n.thin = nt, n.iter = ni,
              n.burnin = nb)

    #Save the Summaries and Samples for later use
    assign(paste0("summ", "_",species[s], "_" ,rgns[r]),
           as.data.frame(m$summary))
    assign(paste0("samp", "_",species[s], "_" ,rgns[r]),
           as.data.frame(rbind(
         m$samples[[1]][,c("beta.time", "beta.PHDI", "beta.int")],
         m$samples[[2]][,c("beta.time", "beta.PHDI", "beta.int")],
         m$samples[[3]][,c("beta.time", "beta.PHDI", "beta.int")]
```

```
        )))

    ##############
    #Calculate Bayes-p
    ##############

    pi <- rbind(m$samples[[1]][,1:55], m$samples[[2]][,1:55],
                m$samples[[3]][,1:55])

    y.ppd <- matrix(NA, nrow = nrow(), ncol = 55)
    bayes.p <- 0
    n.iter <- nrow(pi)
    for(k in 1:n.iter){

      y.ppd[k,] <- rbinom(55,species_rgn[,3],prob = pi[k,])

      ### Calculate Deviance Scores
      score1 <- -2*sum(dbinom(species_rgn[,1], species_rgn[,3],
                    pi[k,], log=T))
      score2 <- -2*sum(dbinom(y.ppd[k,], species_rgn[,3], pi[k,],
                    log=T))

      ### Add to bayes p
      if(score1 >= score2){
        bayes.p <- bayes.p + 1
      }

    }

    ### Calculate Bayes p-value
    assign(paste0(species[s], "_" ,rgns[r],"_pval"),
           bayes.p/n.iter)

  }

}
```

6 Figures

6.1 Release Plots

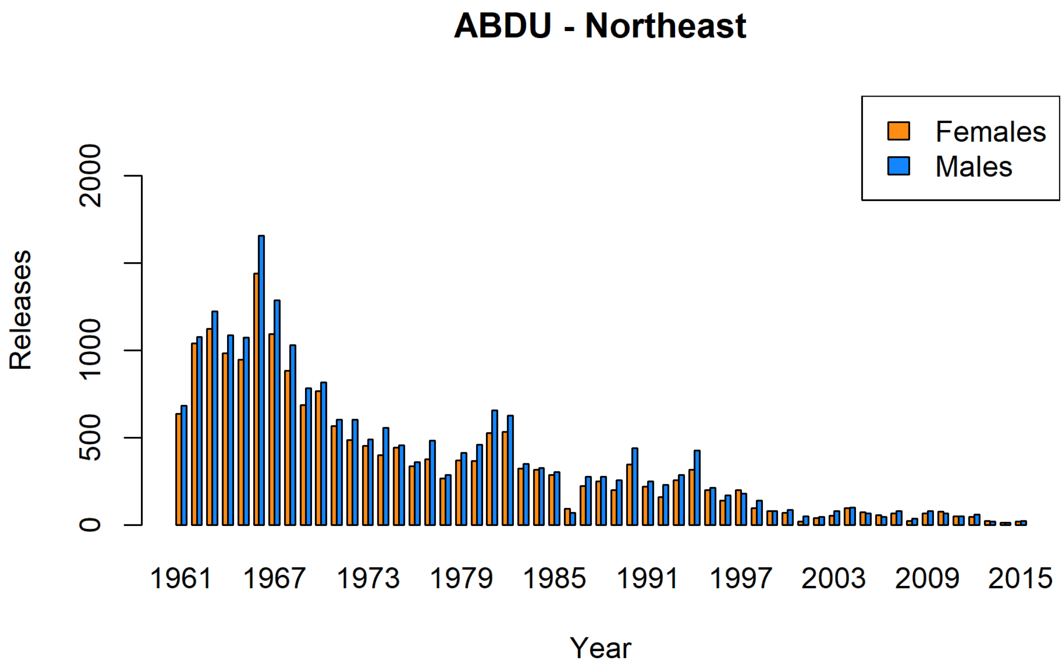

Figure 1: Total releases of American black ducks (abdu) in the Northeast (NE) region from 1961-2015 by females (orange) and males (blue).

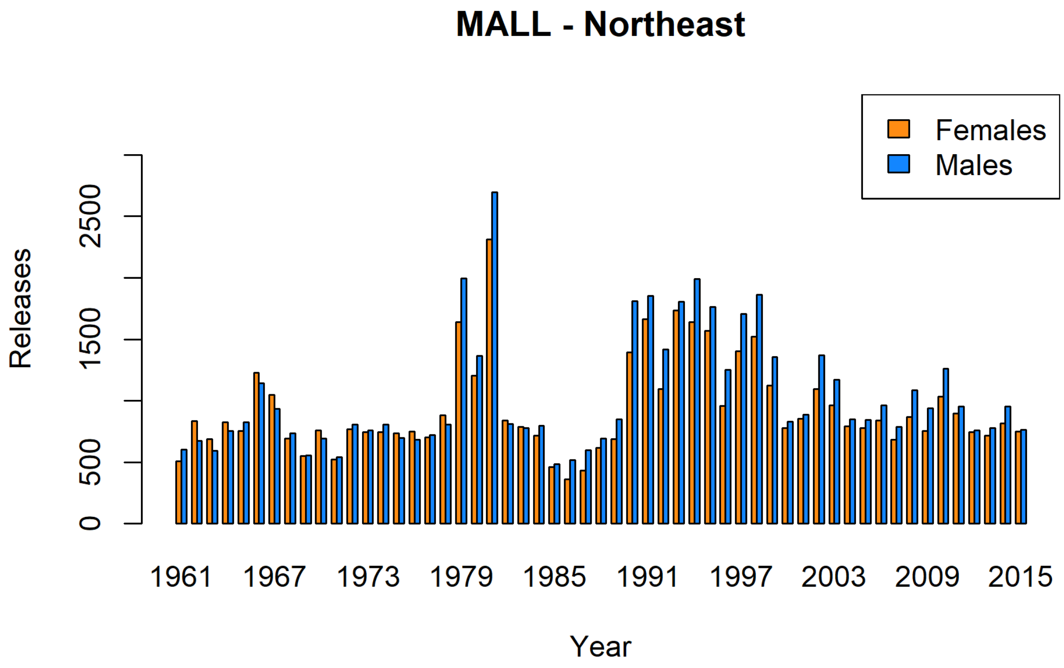

Figure 2: Total releases of mallards (mall) in the Northeast (NE) region from 1961-2015 by females (orange) and males (blue).

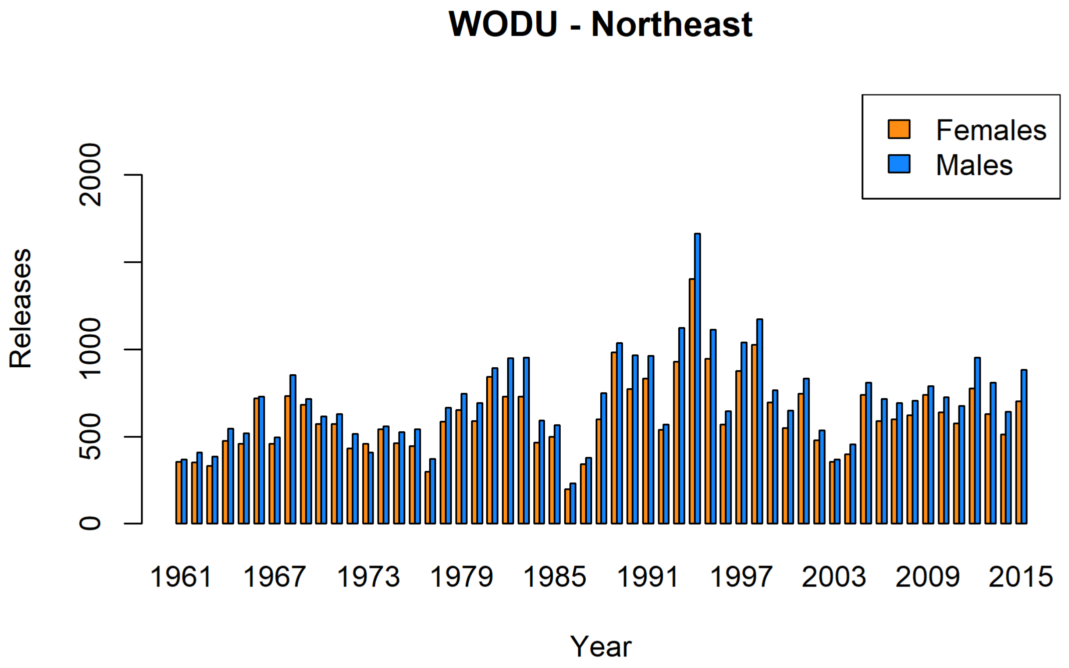

Figure 3: Total releases of wood ducks (wodu) in the Northeast (NE) region from 1961-2015 by females (orange) and males (blue).

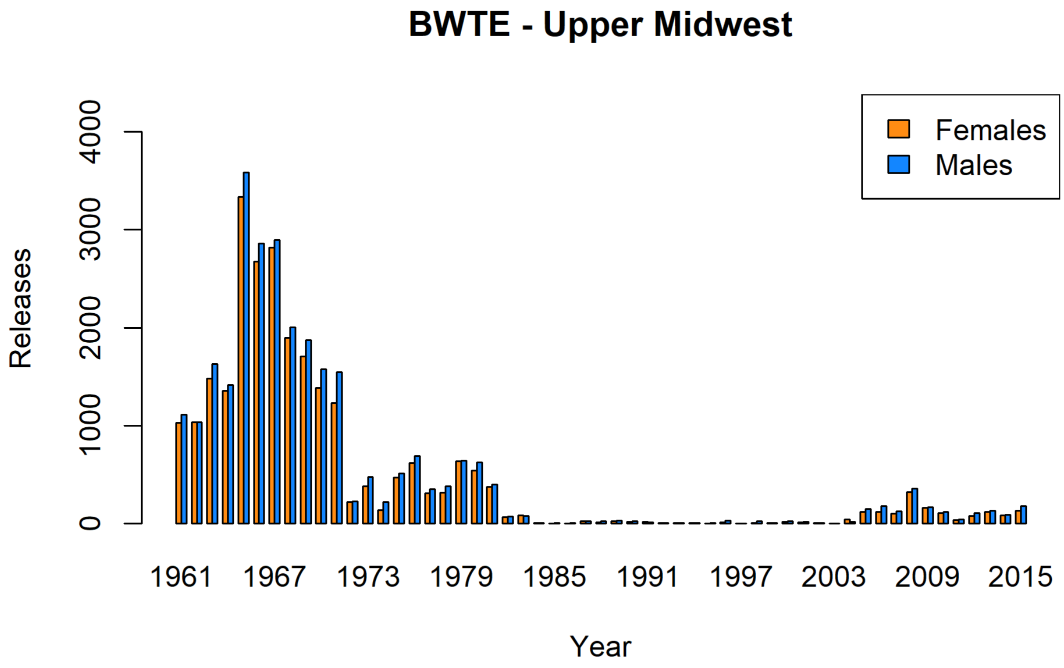

Figure 4: Total releases of blue-winged teal (bwte) in the Upper Midwest (UM) region from 1961-2015 by females (orange) and males (blue).

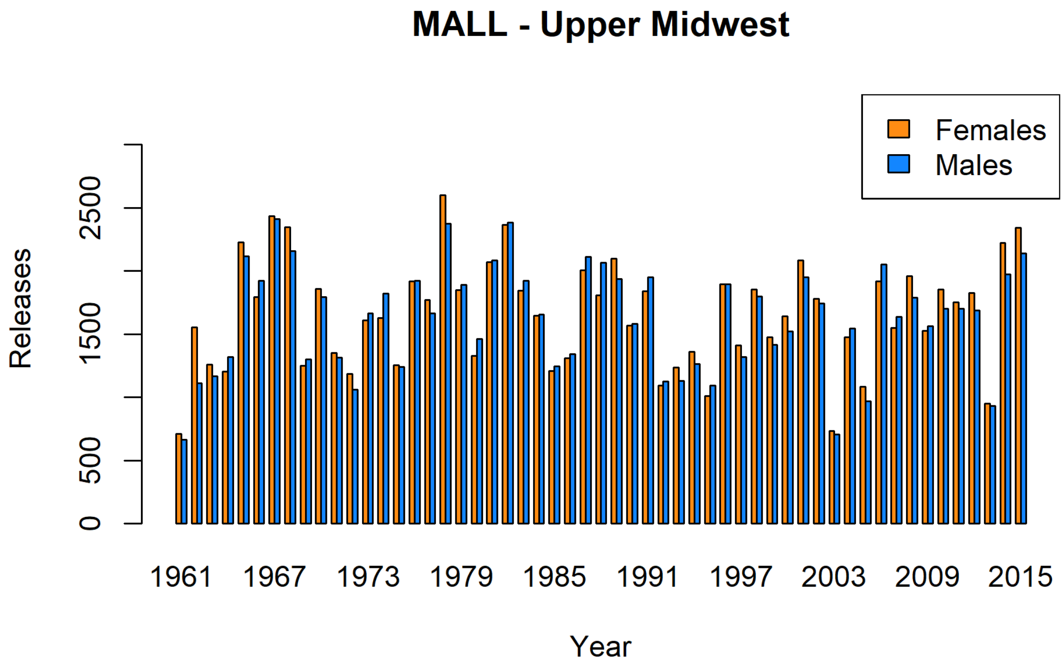

Figure 5: Total releases of mallards (mall) in the Upper Midwest (UM) region from 1961-2015 by females (orange) and males (blue).

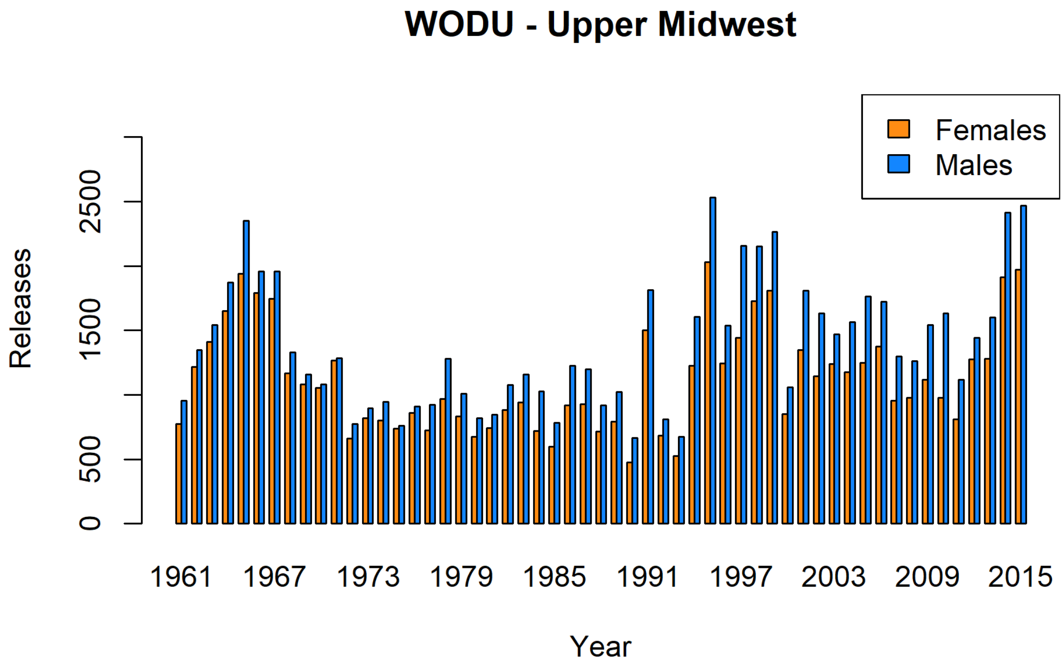

Figure 6: Total releases of wood ducks (wodu) in the Upper Midwest (UM) region from 1961-2015 by females (orange) and males (blue).

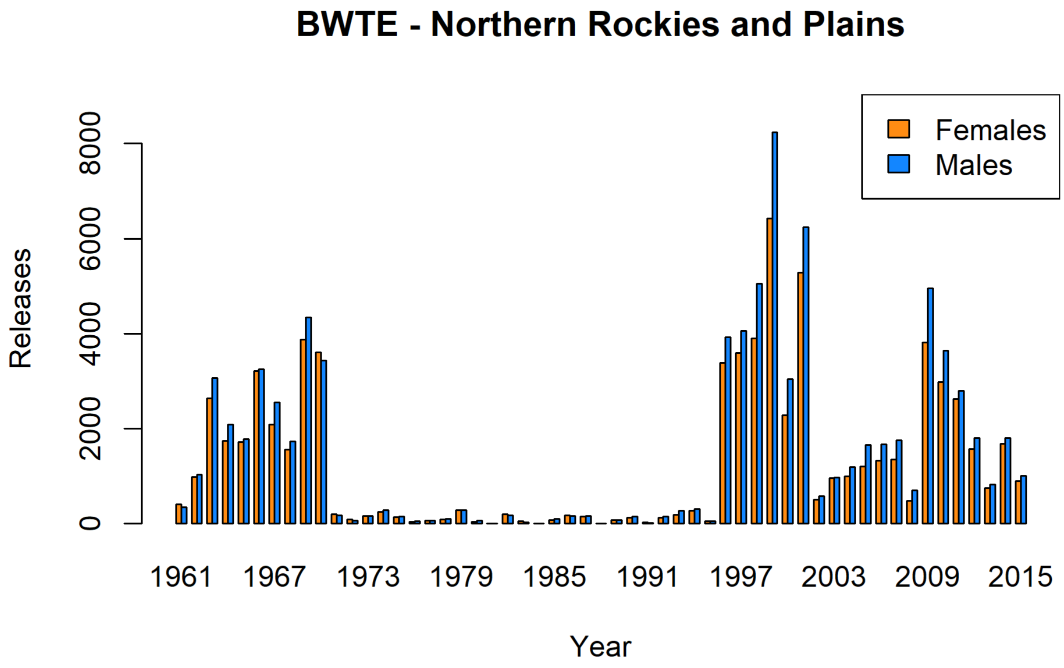

Figure 7: Total releases of blue-winged teal (bwte) in the Northern Rockies and Plains (RP) region from 1961-2015 by females (orange) and males (blue).

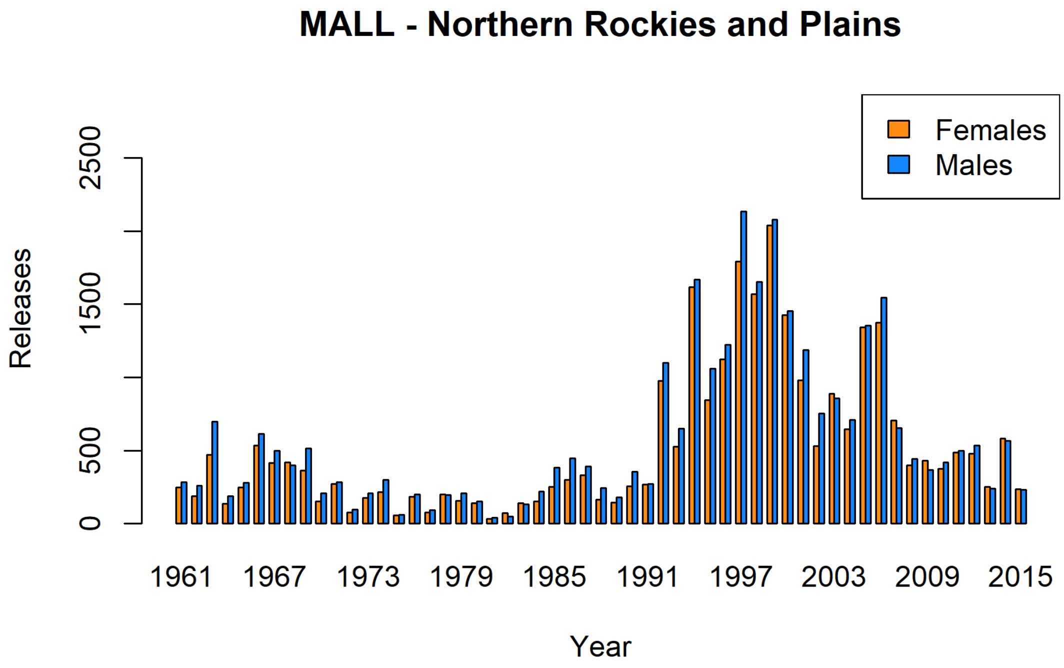

Figure 8: Total releases of mallards (mall) in the Northern Rockies and Plains (RP) region from 1961-2015 by females (orange) and males (blue).

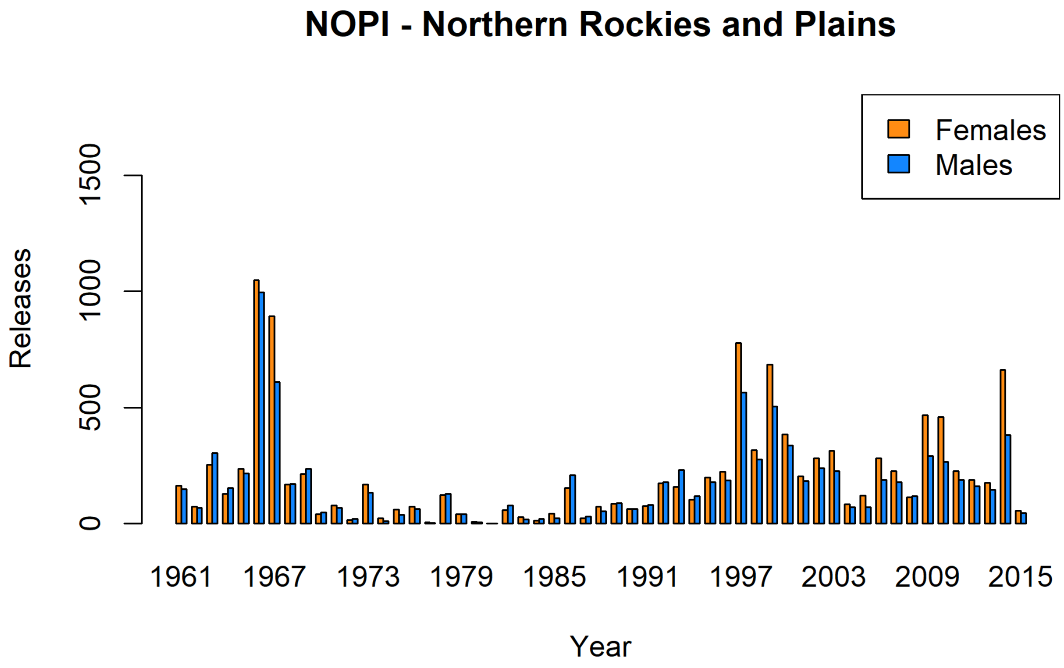

Figure 9: Total releases of northern pintails (nopi) in the Northern Rockies and Plains (RP) region from 1961-2015 by females (orange) and males (blue).

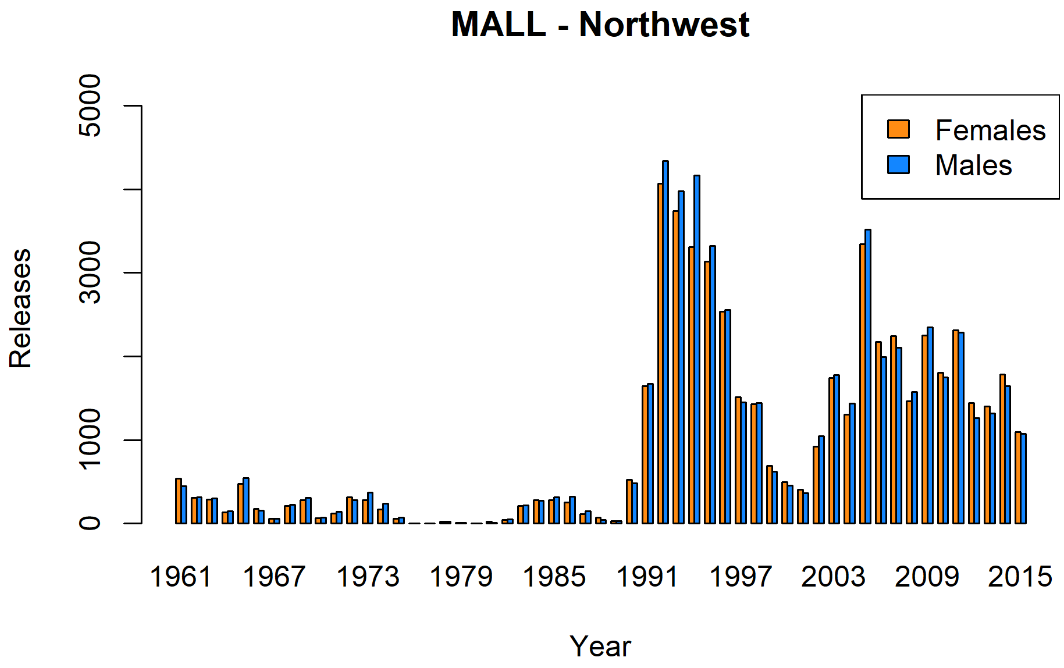

Figure 10: Total releases of mallards (mall) in the Northwest (NW) region from 1961-2015 by females (orange) and males (blue).

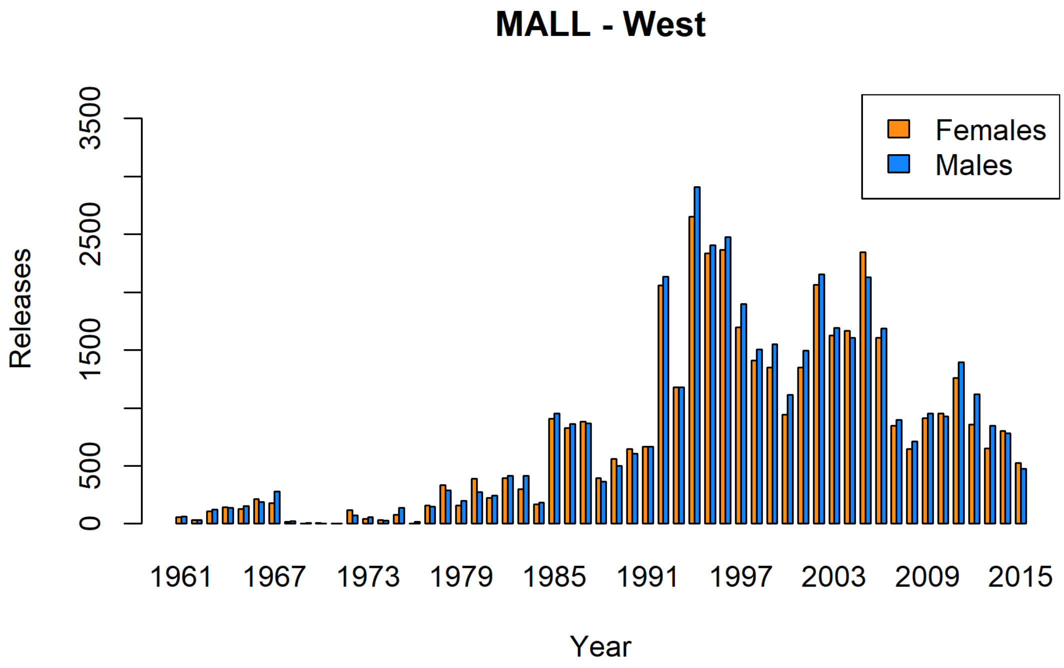

Figure 11: Total releases of mallards (mall) in the West (WE) region from 1961-2015 by females (orange) and males (blue).

6.2 Sex Ratio Plots

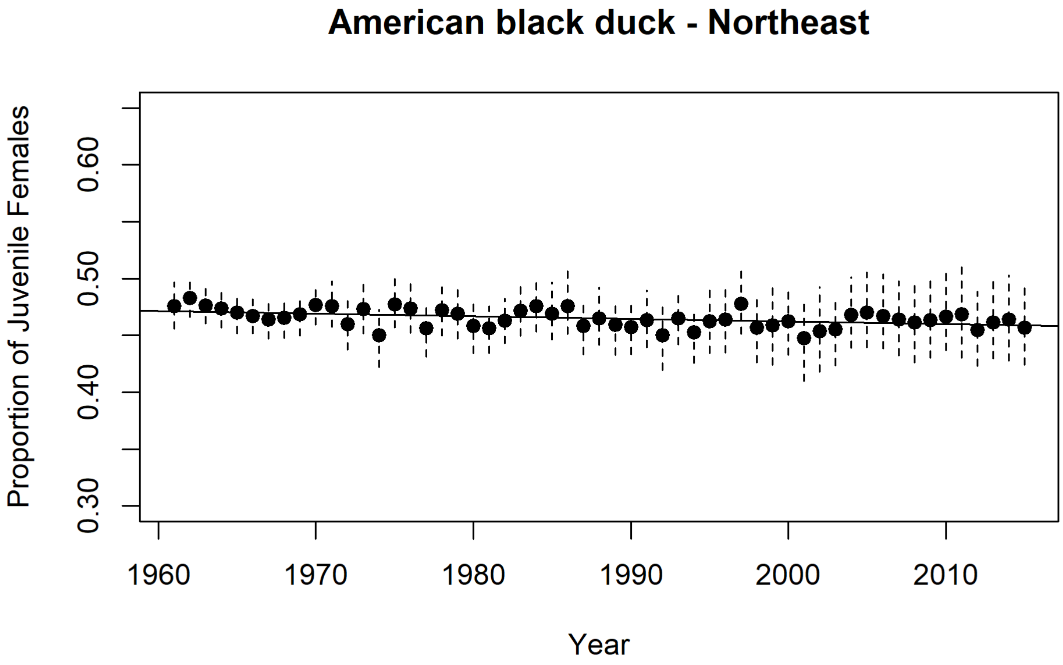

Figure 12: Proportion of female juvenile American black ducks (ABDU) in the Northeastern(NE) region. Regions are by the U.S. Climate Regions (Karl and Koss, 1984) and release data comes from the Bird Banding Laboratory.

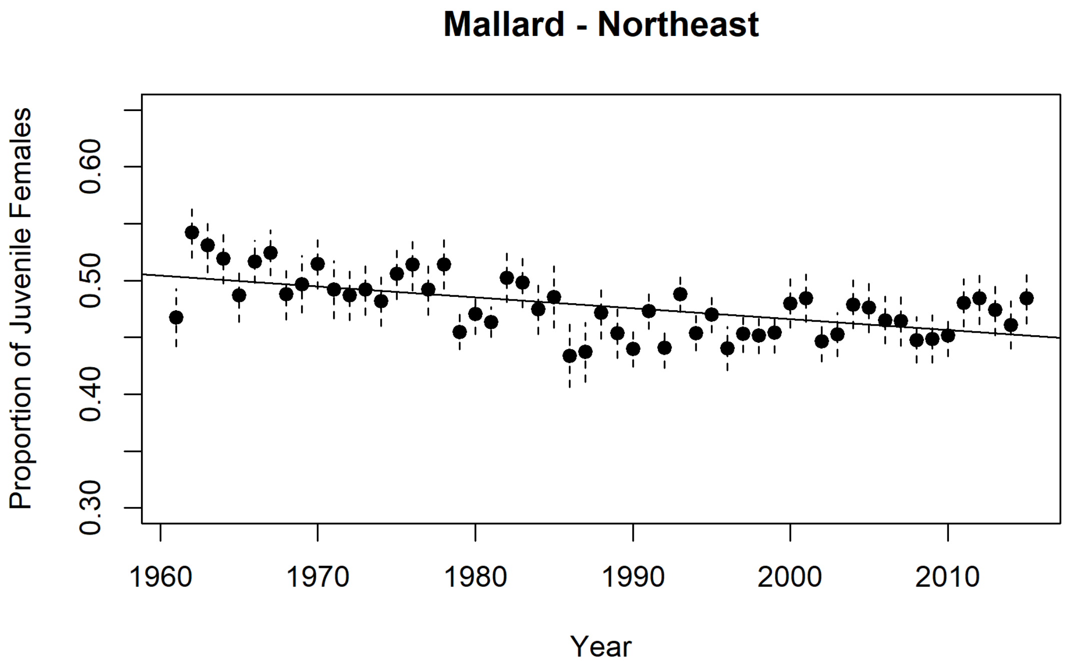

Figure 13: Proportion of female juvenile mallards (MALL) in the Northeastern region. Regions are by the U.S. Climate Regions (Karl and Koss, 1984) and release data comes from the Bird Banding Laboratory.

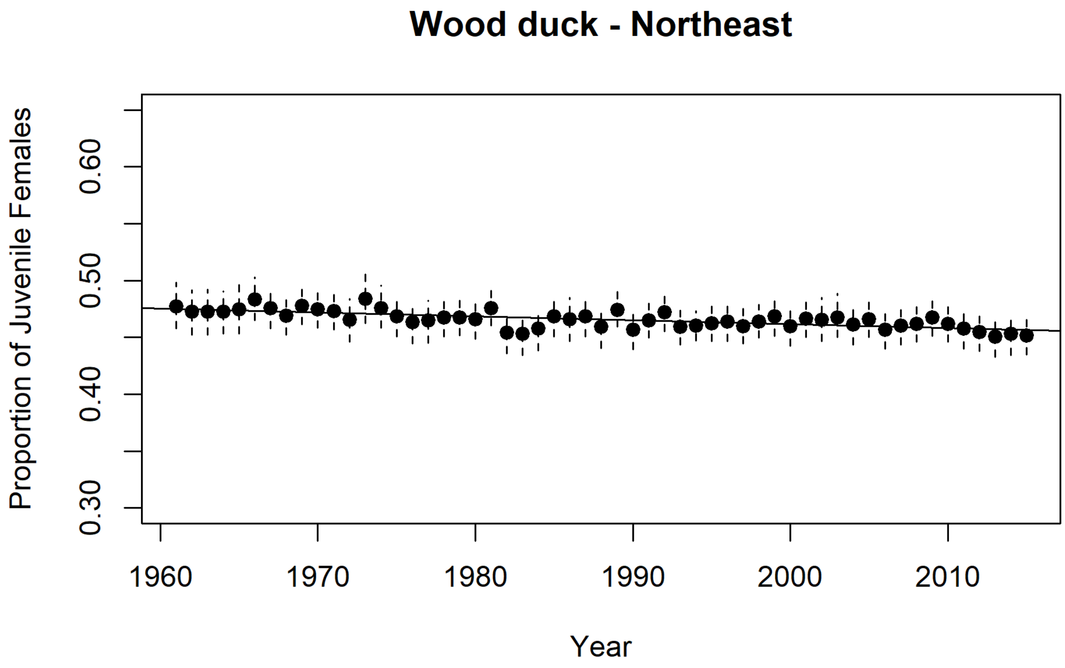

Figure 14: Proportion of female juvenile wood ducks (WODU) in the Northeastern region. Regions are by the U.S. Climate Regions (Karl and Koss, 1984) and release data comes from the Bird Banding Laboratory.

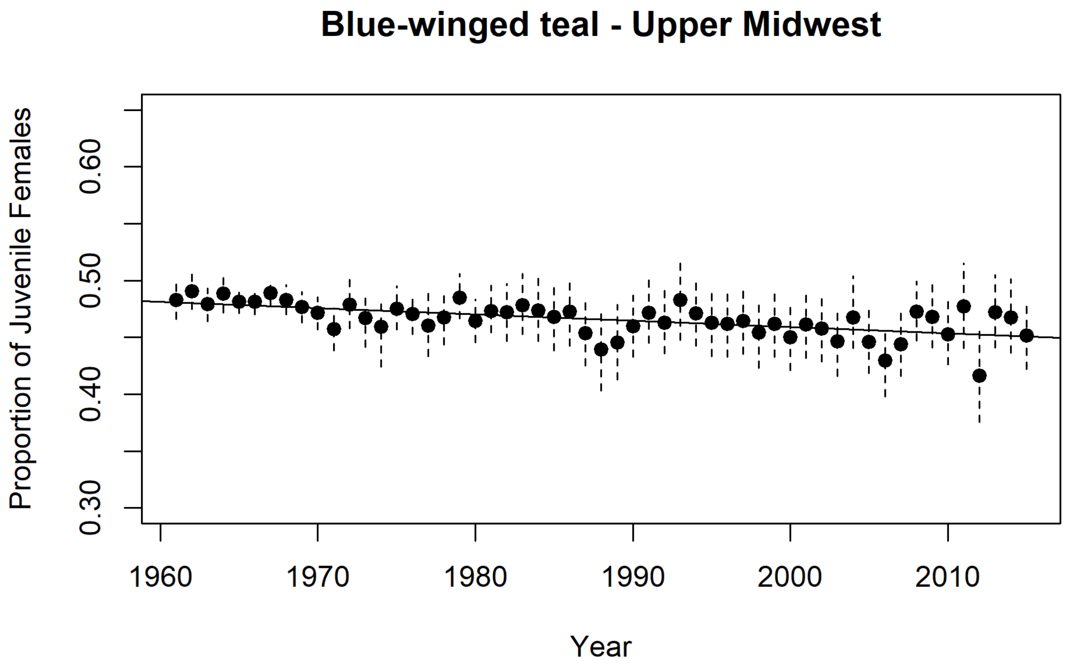

Figure 15: Proportion of female juvenile blue-winged teals (BWTE) in the Upper Midwest region. Regions are by the U.S. Climate Regions (Karl and Koss, 1984) and release data comes from the Bird Banding Laboratory.

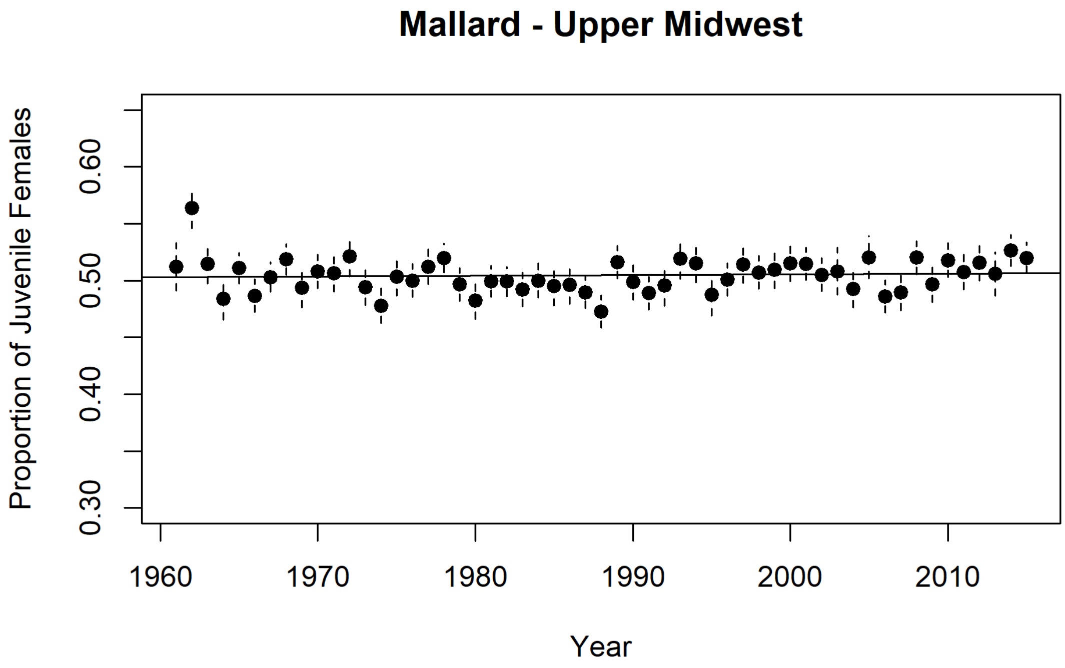

Figure 16: Proportion of female juvenile mallards (MALL) in the Upper Midwest region. Regions are by the U.S. Climate Regions (Karl and Koss, 1984) and release data comes from the Bird Banding Laboratory.

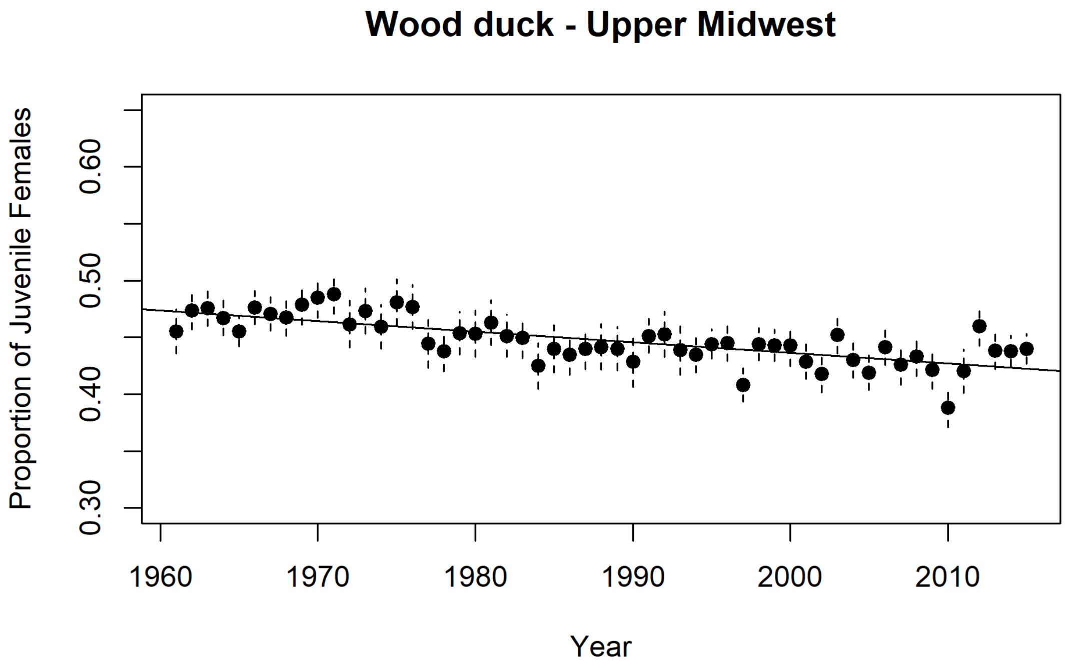

Figure 17: Proportion of female juvenile wood ducks (WODU) in the Upper Midwest region. Regions are by the U.S. Climate Regions (Karl and Koss, 1984) and release data comes from the Bird Banding Laboratory.

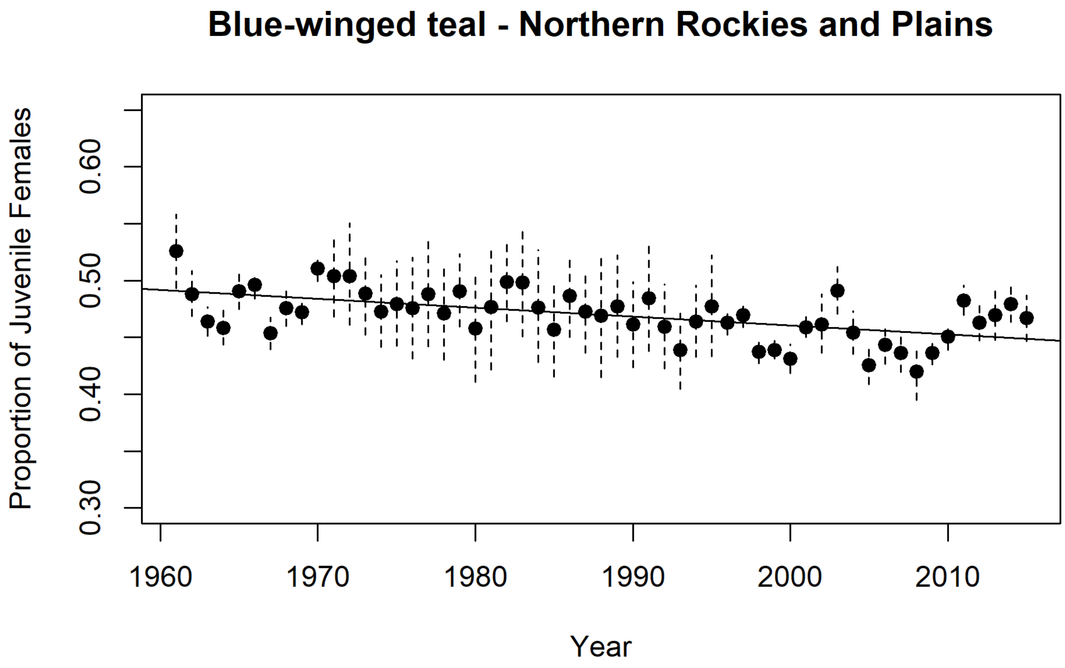

Figure 18: Proportion of female juvenile blue-winged teals (BWTE) in the Northern Rockies and Plains region. Regions are by the U.S. Climate Regions (Karl and Koss, 1984) and release data comes from the Bird Banding Laboratory.

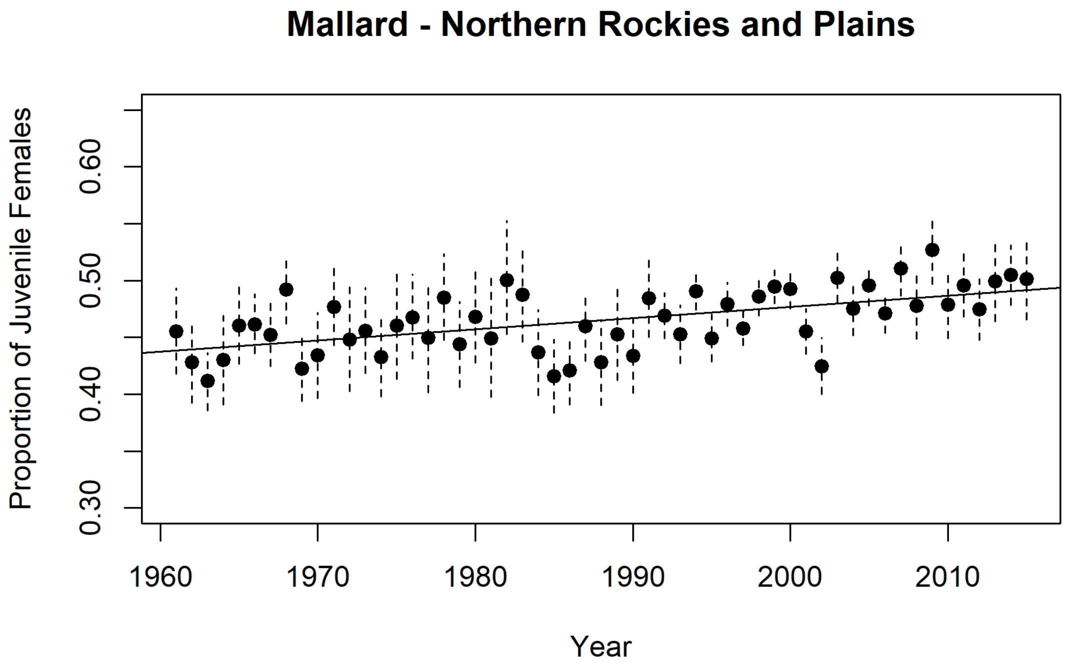

Figure 19: Proportion of female juvenile mallards (MALL) in the Northern Rockies and Plains region. Regions are by the U.S. Climate Regions (Karl and Koss, 1984) and release data comes from the Bird Banding Laboratory.

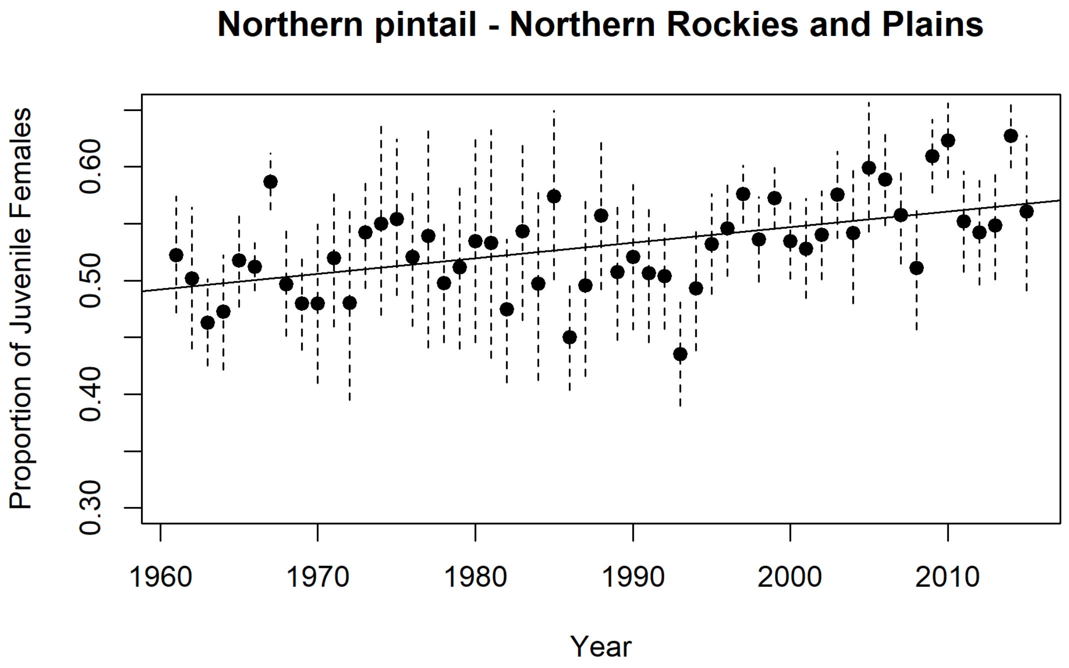

Figure 20: Proportion of female juvenile northern pintails (NOPI) in the Northern Rockies and Plains region. Regions are by the U.S. Climate Regions (Karl and Koss, 1984) and release data comes from the Bird Banding Laboratory.

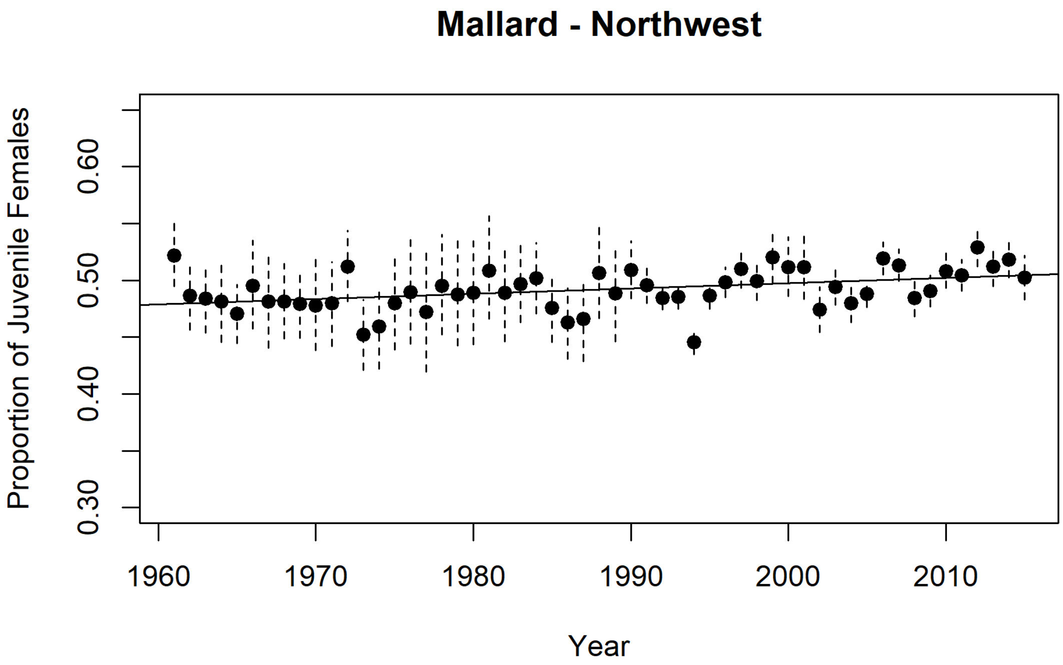

Figure 21: Proportion of female juvenile mallards (MALL) in the Northwest region. Regions are by the U.S. Climate Regions (Karl and Koss, 1984) and release data comes from the Bird Banding Laboratory.

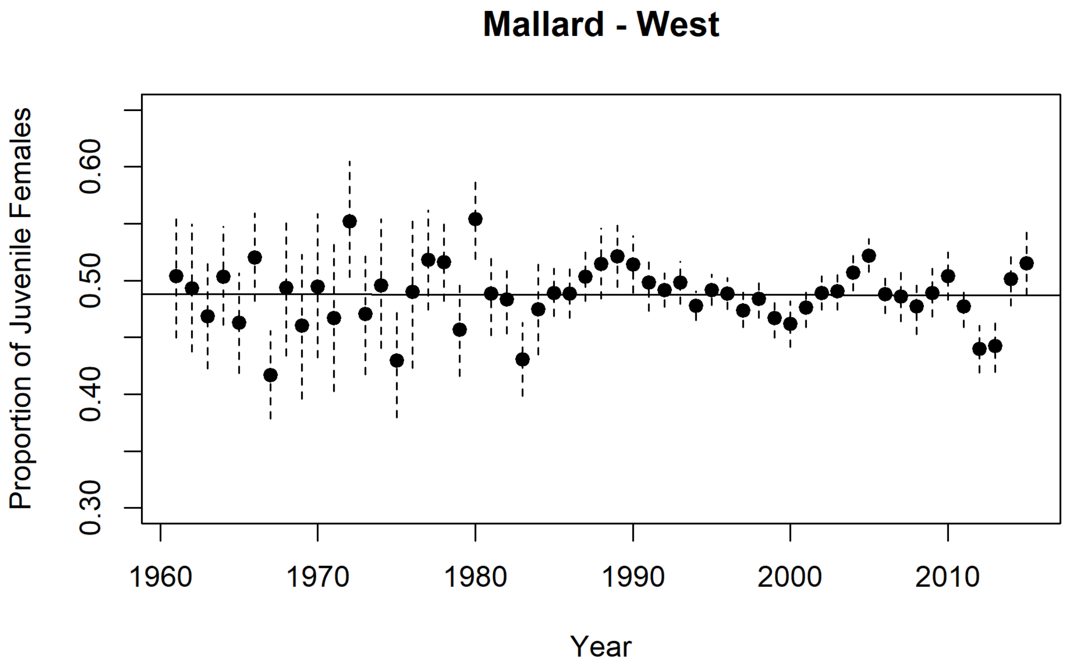

Figure 22: Proportion of female juvenile mallards (MALL) in the West region. Regions are by the U.S. Climate Regions (Karl and Koss, 1984) and release data comes from the Bird Banding Laboratory.
